# A Review of Carbon-Based Materials for Safe Lithium Metal Anodes

**DOI:** 10.3389/fchem.2019.00721

**Published:** 2019-11-04

**Authors:** Yan Liu, Xifei Li, Linlin Fan, Shufeng Li, Hirbod Maleki Kheimeh Sari, Jian Qin

**Affiliations:** ^1^School of Materials Science and Engineering, Institute of Advanced Electrochemical Energy, Xi'an University of Technology, Xi'an, China; ^2^Shaanxi International Joint Research Center of Surface Technology for Energy Storage Materials, Xi'an, China; ^3^State Center for International Cooperation on Designer Low-Carbon & Environmental Materials (CDLCEM), Zhengzhou University, Zhengzhou, China

**Keywords:** lithium metal anodes, dendrite, electrochemical performance, carbon-based materials, energy storage

## Abstract

Lithium metal is a promising anode material with extremely high theoretical specific capacity (3,860 mA h g^−1^), low density (0.59 g cm^−3^), and the lowest negative electrochemical potential of all potential candidates (−3.04 V vs. the standard hydrogen electrode). However, uncontrollable Li dendrite growth leads to a short lifespan and catastrophic safety hazards, which has restricted its practical application for many years. Some effective strategies have been adopted regarding these challenges, including electrolyte modification, introducing a protective layer, nanostructured anodes, and membrane modification. Carbon-based materials have been demonstrated to significantly address the challenge of Li dendrites. In this review, carbon-based materials and their application and challenges in lithium metal anode protection have been discussed in detail. In addition, the applications of lithium anodes protected by carbon-based materials in Li-S batteries and Li-O_2_ batteries have been summarized.

## Introduction

Environmental pollution has become a serious issue, and green energies, including batteries, particularly rechargeable lithium-metal batteries, have received extensive attention from researchers. Lithium metal is the ultimate choice for the anode in lithium batteries due to it having the highest theoretical capacity (3,860 mA h g^−1^ or 2,061 mA h cm^−3^) and lowest electrochemical potential (−3.04 V vs. the standard hydrogen electrode) (Tarascon and Armand, 2001; Wu et al., 2014) among all the possible candidates (Lin et al., 2017).

However, growth of Li dendrites during repeated charge/discharge processes and low Coulombic efficiency (CE) (Wu et al., 2014; Maleki Kheimeh Sari and Li, 2019) lead to early cell death, rapid cycling capacity decay, and catastrophic thermal runaway (Yingying et al., 2014; Zheng et al., 2014; Kai Z. et al., 2016; Zhang R. et al., 2016), which substantially impede the development of lithium metal batteries (LMBs). Hence, although the theoretical capacity of a lithium metal anode is about ten times greater than that of a graphite anode (372 mA h g^−1^), it has not yet been commercialized. Furthermore, compared to lithium-ion batteries (LIBs, specific energy ~250 Wh kg^−1^), Li-S and Li-O_2_ systems can further boost specific energies to up to ~650 and 950 Wh kg^−1^ (Lin et al., 2017), respectively. Therefore, it can be speculated that in the near future lithium metal anodes will be an indispensable part of Li-S, Li-O_2_, and Li-CO_2_ batteries, realizing a high specific capacity to fulfill the ever-growing energy density requirements of portable electronic devices and electrical vehicles (Bruce et al., 2011; Aurbach et al., 2016; Zhi et al., 2016; Lim et al., 2017). The only important barrier to this development is the growth of lithium dendrites, which needs to be curtailed.

Certain methods have been adopted in this regard, including using LiX alloys (X = Al, B, Si, Sn, C, etc.), organic electrolyte and Li metal/electrolyte interface modifications, solid-state electrolytes, and structured anode designs (Cheng et al., 2015), leading to some satisfactory results. Notably, many researchers have used carbon-based materials to protect lithium metal anodes and have made significant progress due to their advantageous characteristics, including superior thermal and electrical conductivity, high temperature resistance, excellent chemical stability, good mechanical strength, and satisfactory lubrication performance.

In this review, we first elaborate on the formation mechanism of lithium dendrites and then summarize the application of carbon-based materials and their composites in lithium metal anode protection. The targeted carbon-based materials include C_60_, carbon nanotubes (CNT), graphene, graphite, and graphdiyne (GDY), which was first synthesized by Li's group in 2010 (Guoxing et al., 2010). The aforementioned graphite-family materials and their composites deliver perfect electrochemical performance, which makes them one of the best options for lithium metal anode protection. The review then goes on to discuss the potential applications and challenges of lithium metal anodes protected by carbon-based materials in Li-S and Li-O_2_ batteries in general.

## The Formation Mechanism of Li Dendrites

**Figure 1:**
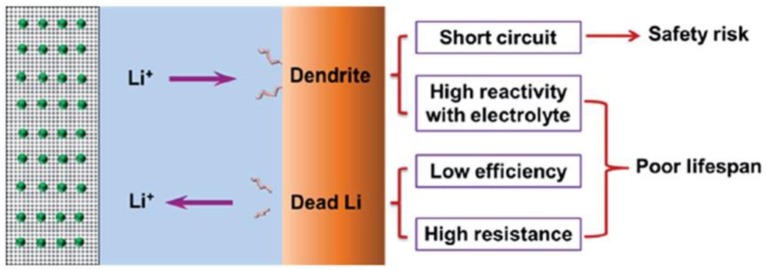
Schematic diagram of the typical morphology of Li dendrites and the related safety risks and lifespan effects. Reprinted with permission from Cheng et al. (2015). Copyright (2015) Royal Society of Chemistry.

The dendritic growth of Li metal has been investigated since the 1960s, and research on Li metal anodes has continued without break during the past 40 years (Suo et al., 2012; Whittingham, 2012; Hong-Jie et al., 2014; Manthiram et al., 2014; Mukherjee et al., 2014; Cheng and Zhang, 2015; Jiulin et al., 2015; Peng et al., 2015). The formation process of lithium dendrites and dead lithium and the related safety risks and lifespan impacts are shown in Figure 1. Although many studies have been devoted to exploring the growth mechanism of these dendrites, its exact nature is still inconclusive. Due to the uneven surface of the lithium sheet, lithium ions are continuously deposited and stripped through charging and discharging; under the influence of space charge, convex areas of the surfacetend to accumulate lithium ions, eventually leading to inhomogeneous deposition of lithium ions and formation of dendrites. The dendrites will pierce the separator, resulting in a short circuit of the cell, which is a potential security issue and can cause a serious accident. Therefore, it is important to understand the formation mechanism of Li dendrites and to find a novel method for restraining it.

There is a widely accepted diffusion model (Equation 1) called the “Sand's time τ,” which is applied to describe the migration properties of lithium ions and electrons (Chazalviel, 1990; Fleury et al., 1990; Brissot et al., 1998; Rosso et al., 2001). Sand's time was proposed for the first time when, Sand (1901) investigated the liberation of hydrogen in a mixture of copper sulfate and sulfuric acid, and it has since been used more generally, including for LMBs.

In the LMB system, the time at which lithium dendrites begin to grow is called “Sand's time.”

(1) $$\begin{array}{r} \tau =\pi D{\left(\frac{eC_{0}}{2Jt_{a}}\right)}^{2} \end{array}$$

In Equation (1), D is the ambipolar diffusion coefficient, *D* = (μ_a_*D*_c_ + μ_c_*D*_a_)/(μ_a_ + μ_c_), where *D*_c_ and *D*_a_ are cationic and anionic diffusion coefficients, *e* is the electronic charge, *C*_0_ is the initial concentration of Li salt, μ_a_ and μ_c_ are anionic and cationic mobilities, respectively, *J* is the effective electrode current density, and *t*_a_ is the anionic transport number *t*_a_ = μ_a_/(μ_a_ + μ_c_).

**Figure 2:**
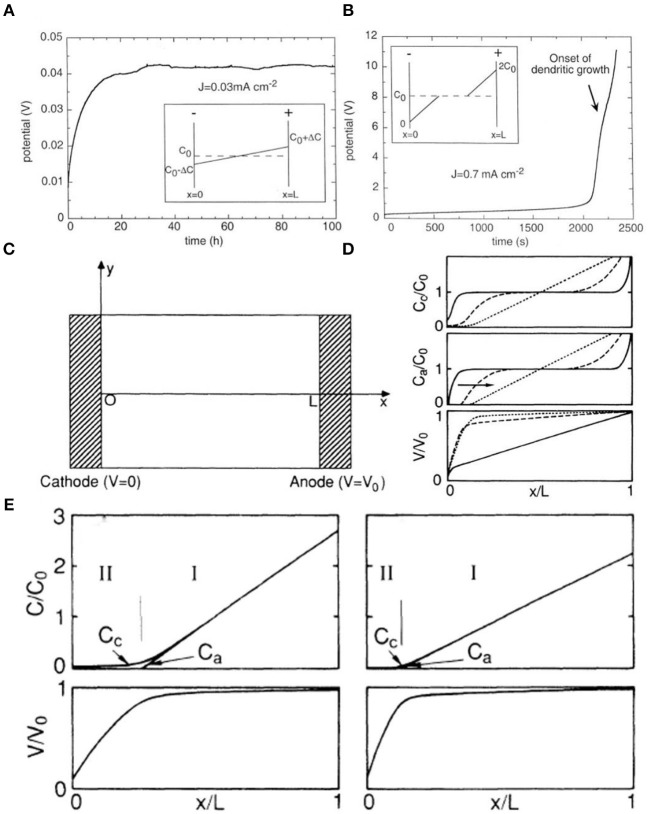
For a given distance L between the electrodes, **(A)** if *J* < J^*^, the system evolves to a steady state where the concentration varies linearly from C_0_ to ΔC at the negative electrode, **(B)** if *J* < J^*^ (semi-infinite approximation), the ionic concentration drops to zero and the cell potential diverges at Sand's time. Here, Sand's time is about 2,100 s [note the different time scales in **(A,B)**]. The inflection on the *V* (*t*) curve shown by the arrow corresponds to the onset of dendritic growth. Reprinted with permission from Brissot et al. (1999). Copyright (1999) Elsevier Science BV. **(C)** Schematic diagram of the cell. **(D)** Profile of the ion concentrations and electrostatic potentials as a function of time. **(E)** Profile of the ion concentrations C_c_ and C_a_ and the electrostatic potential V resulting from the numerical simulation in the hypothetical case of uniform deposition with negligible growth of the cathode (C_0_ = 10^10^ cm^−3^). **(D)** Profile of the ion concentrations C_c_ and C_a_ and the electrostatic potential V resulting from the numerical simulation in the hypothetical case of uniform deposition with negligible growth of the cathode (C_0_ = 10^11^ cm^−3^). Reprinted with permission from Chazalviel (1990). Copyright (1990) American Physical Society.

In the “Sand's time” model, *J* and *t*_*a*_ are in inverse proportion to τ. If *J* or *t*_*a*_ decreases and the other parameters remain constant, Sand's time (τ) gets larger, which indicates that the cell has a long lifespan before the growth of Li dendrites (Zhang R. et al., 2016). Equation (2) can predict two different behaviors for a symmetrical cell (Brissot et al., 1999). One is when δC/δx <2C_0_/L (Figure 2A), in which case the ionic concentration profile evolves to a steady state at which the concentration gradient is constant (Bruce and Vincent, 1987) and the voltage is also stable. The other is when δC/δx >2C_0_/L (Figure 2B), in which case the concentration will evolve to reach zero at the negative electrode at Sand's time (Brissot et al., 1999). Meanwhile, the voltage becomes very unstable until it eventually drops to zero at Sand's time due to the ionic concentrations in the vicinity of the negative electrode (Sand, 1901). If the current density (*J*) is greater than the critical value (J^*^), the aforementioned phenomenon will appear; Equation (3) represents the critical value J^*^. The concentration of cations and anions will then endure a dramatic change, resulting in the aggregation of a large amount of positive charge in the negative electrode, which leads to a local space charge (Brissot et al., 1999).

(2) $$\begin{array}{r} \frac{\partial C}{\partial x}\left(x=0\right)=\frac{-J}{eD\left(1+\frac{\mu_{a}}{\mu_{c}}\right)} \end{array}$$

(3) $$\begin{array}{r} J^{\text{*}}\text{=}\frac{2eC_{0}D}{t_{a}L} \end{array}$$

The theory of space charge was put forward by Fleury et al. (1990) and can explain the growth process of lithium dendrites. A thin rectangular cell model was constructed by Chazalviel in a dilute solution under a strong electric field (Figure 2C). Chazalviel found that diffusion and migration play a dominant role in different regions of the rectangular cell and calculated the distributions of potential and of ion concentration (Figure 2E). Region I (Z_c_C_c_ ≈ Z_a_C_c_) is called the quasi-neutral region, where the electrostatic potential is close to zero and V_0_ at x = 0 and x = L, respectively. The region extends from the anode to the vicinity of the cathode and dominates most areas of the cell, controlled by a diffusion mechanism. Region II (C_a_ < < C_c_) is called the space-charge region, which is near the cathode, occupies a very small fraction of the cell, and is controlled by the migration of the electric field. Indeed, the electron transfer speed is faster than the ion diffusion rate. The ion concentration (C_a_, C_b_) and potential (V) change over time (Figure 2D). The electric field is distributed evenly, and no space charge is generated at *t* = 0. The positive ions migrate from anode to cathode, resulting in congregation of negative ions near the anode. Depletion develops in the vicinity of the cathode, making it a space-charge region. However, the congregation of anions in the anode leads to enhancement of the local electric field and the attraction of more cations, and the intensified electric field results in rapid cation deposition. In fact, lithium ions are being constantly released and embedded during the charging and discharging process, and the charge on the protruding part of the lithium metal surface tends to be inhomogeneously distributed, which generates a space-charge region and consequently dendrites. Hence, dendrite growth can be inhibited by controlling the formation of a space-charge region. This can be done by increasing the convective motions of the electrolyte, which can effectively control the stirring rate, salt solution concentration gradient, and current density.

## Carbon-Based Materials for Safe Lithium Metal Anodes

**Figure 3:**
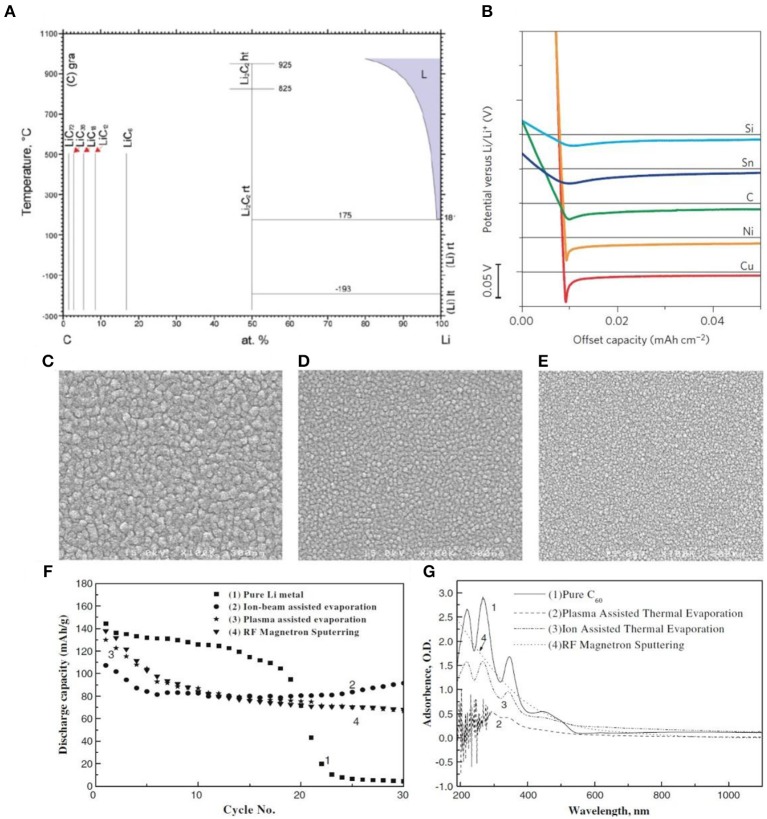
**(A)** The phase diagram of lithium and carbon. **(B)** Shifted voltage profiles of various materials with negligible solubility in Li during Li deposition at a current density of 10 μA cm^−2^. Reprinted with permission from Kai Y. et al. (2016). Copyright (2016b) Springer Nature Limited. SEM images of fullerene C_60_ film deposited by three different techniques **(C)** Ion beam assisted evaporation. **(D)** Plasma assisted evaporation and **(E)** radio frequency magnetron sputtering. **(F)** The cyclic performance of a pure lithium electrode and fullerene C_60_-coated lithium electrodes obtained by three different techniques. **(G)** UV-Vis spectra of the pure C_60_ film and C_60_ coated films obtained by three vacuum techniques. Reprinted with permission from Arie et al. (2008). Copyright (2008) Springer Nature Limited.

Many effective methods have been adopted thus far to inhibit lithium dendrite growth. Among them, researchers have carried out a lot of work using carbon-based materials to protect lithium metal anodes and have made great progress. The interfacial energy between carbon-based materials and Li metal is vital to this process. Li is body-centered cubic (BCC) and belongs to the Im3m space group, while C is categorized into the cubic system (diamond) or hexagonal system (graphite) and belongs to the Fd3m (diamond) space group and P6/mmm (graphite) space group. This mismatch between the two-phase crystal structure increases because of the interface energy. Additionally, their radii are different: the radius of C is 0.077 nm, while that of Li is nearly double that (i.e., 0.152 nm). From the phase diagram of lithium and carbon (Figure 3A), it is evident that lithium and carbon can form an alloy phase while overpotential (about 14 mV) still exists (see Figure 3B); indeed, carbon materials have no solubility in lithium. Moreover, compared with Cu and Ni, the voltage dip of carbon is relatively small, which means that the nucleation overpotential [i.e., the difference between the bottom of the voltage drop and the flat portion of the voltage platform (Kai Y. et al., 2016)] of Li metal is much smaller. In this section, the application of different types of carbon materials, carbon materials modified by doping, and composites with carbon materials in lithium protection are thoroughly reviewed.

### Types of Carbon Materials for Lithium Metal Anode Protection

Based on their morphologies, carbon materials can be categorized into several groups including fullerenes (C_60_), graphite, CNT, graphene, and graphdiyne (GDY). It is well-known that different morphologies can show different physicochemical performances. In this section, the application of carbon materials of different types for lithium metal protection is introduced.

#### Fullerenes (C_60_)

In 1985, fullerenes were firstly discovered by scientists at Ross University in Texas. There are 60 C atoms in a C_60_ molecule, which form 32 planes, 20 regular hexagons, and 12 regular pentagons. The fullerene structure is similar to that of graphite. Graphite is a graphene layer composed of six-membered rings, whereas fullerene contains five-, six-, and seven-membered rings. In addition, the carbon atoms in fullerenes are bonded together in a spherical dome structure. It can be classified among molecular crystals with a low melting point, low hardness, and insulative properties. If they suffer from high-intensity photon or electron irradiation or interact with plasma (Arie et al., 2008), fullerenes can evolve from the ground state and form polymeric materials. Indeed, chemically modified synthetic fullerenes have been used by some researchers in the field of electrochemistry (Giacalone and Nazario, 2006). Interestingly, Arie et al. (2008) adopted three methods (i.e., radio frequency magnetron sputtering, plasma, and ion-assisted thermal evaporation) to coat a Li metal surface with fullerenes. The results show that the C_60_ film formed through ion-assisted thermal evaporation is the thickest (Figure 3C), that from plasma-assisted evaporation is moderate (Figure 3D), and that from magnetron sputtering is the finest (Figure 3E). As observed in Figure 3F, coating the surface of lithium metal with a carbon film can yield better cycle stability compared to pure lithium metal. It can be seen from Figure 3G that the absorbance plot of the carbon film resulting from the ion-beam assisted technique shows a similarity to that of pure C_60_. Both of them show three peaks in the 200 to 400 nm wavelength range. The spectrum provided by the ion beam case shows smaller peaks in comparison to that of pure C_60_, indicating that this carbon film may contain a portion of initial fullerene C_60_. Thus, the C_60_-coated lithium electrode obtained by ion-assisted thermal evaporation displayed the best electrochemical performance.

#### Carbon Nanotubes (CNTs)

**Figure 4:**
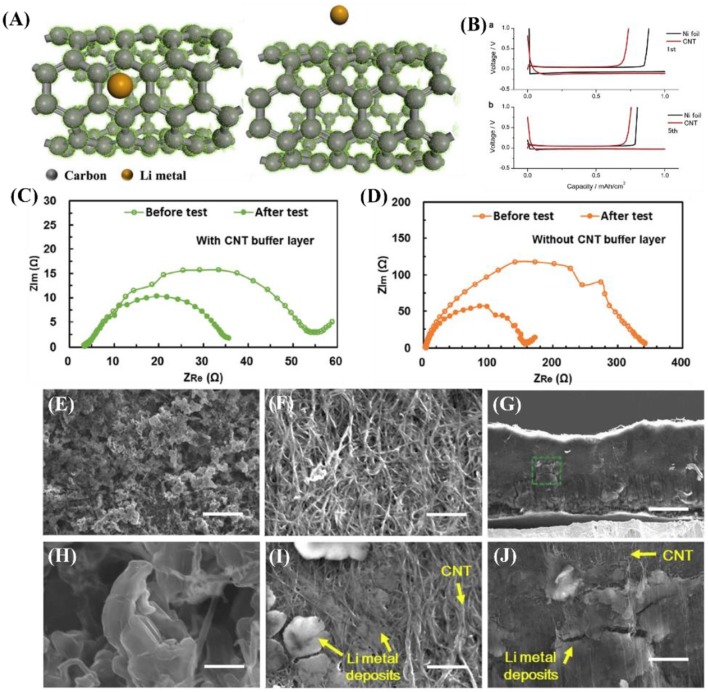
**(A)** Top view and cross-section view of Li atom binding with carbon atom of CNT calculated with DFT using the CASTEP code in Materials Studio, Accelrys Inc. (version 2017 R2). The local potential of CNT is shown as green dots. Reprinted with permission from Zhang H. et al. (2018). Copyright (2018) Springer Nature Limited. **(B)** Voltage profiles of electrochemical cells with Ni foil (black curve) or CNT matrix (red curve) as the substrate material during Li-metal deposition/dissolution for the (top) 1st and (bottom) 5th cycle at a current density of 1.0 mA cm^−2^ and capacity of 1.0 mA h cm^−2^. EIS curves at frequencies ranging from 100 kHz to 100 mHz of the symmetric battery **(C)** with and **(D)** without the CNT buffer layer before the test and after 40 charge–discharge cycles. Reprinted with permission from Zhang D. et al. (2016). Copyright (2016b) Royal Society of Chemistry. **(E,H)** Top-view SEM images of Ni foil taken out from the electrochemical cell after the 5th Li-metal deposition process. **(F,I)** Top-view and **(G,J)** side-view SEM images of CNT matrix taken out from the electrochemical cell after the 5th Li-metal deposition process. The scale bar is 20 mm in **E,G**, and 2 mm in **F,H–J**. Reprinted with permission from Matsuda et al. (2017). Copyright (2017) Elsevier Science BV.

Carbon nanotubes possess good mechanical properties, good adhesion, and excellent electrical conductivity, thereby improving the interface properties (Wang et al., 2013). Moreover, because of their large internal space and specific surface area, CNTs can alleviate the volume expansion of LMBs during continuous deposition/dissolution. Meanwhile, the binding energy between lithium and CNT is 1.19 eV (Figure 4A), which is lower than that with copper (2.57 eV), graphene (3.64 eV), and N (4 eV) (Zhang H. et al., 2018). CNTs have smaller R_SEI_ than the pristine lithium, resulting from the better wettability of the interface between the electrode surface and the electrolyte. As shown in Figures 4C,D, in the Zhang D. et al. (2016) study, the resistance of the battery with a CNT buffer layer was 50 Ω before the test, while the resistance of the battery without a CNT buffer layer was 320 Ω. It is obvious that the interfacial resistance of the battery with the buffer layer was smaller and more stable, which indicates higher interfacial stability and better electrode-electrolyte contact. This excellent electrode wettability is advantageous for facilitating diffusion of the electrolyte into the inner space of the electrode, thus reducing the transfer resistance due to the reduced ion diffusion length during charge and discharge (Bai et al., 2018). Therefore, the better wettability of CNTs at the electrode surface and electrolyte interface can be observed through electrochemical impedance spectroscopy (EIS). Thus, CNT is considered to be a promising three-dimensional substrate material for lithium metal anodes.

Nakanishi's group used Ni foil and a CNT matrix as the working electrode and lithium foil as the reference electrode (Matsuda et al., 2017). As shown in Figure 4B, the 1st and 5th cycles at a current density of 1.0 mA cm^−2^ and capacity of 1.0 mA h cm^−2^ revealed that the CNT matrix has superior electrochemical stability compared to Ni foil. This is because a more stable solid electrolyte membrane is formed in the CNT matrix during continuous deposition/dissolution. According to Figure 4E, the micrometer-sized Li-metal deposits are evenly distributed on the surface of the Ni foil, but the magnified SEM shows dendritic growth (Figure 4H). In contrast, the surface of the CNTs is smooth, without any dendrite formation (Figure 4G). The lithium deposit is clearly observable in the SEM images in Figure 4I (top-view) and Figures 4G,J (side view).

**Figure 5:**
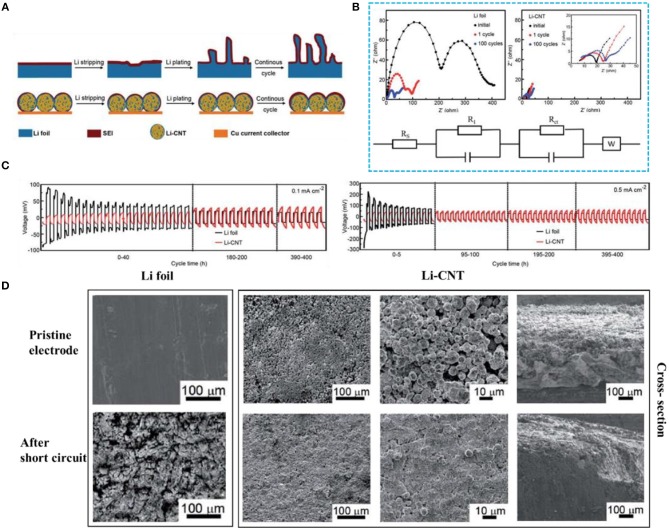
**(A)** Li stripping and plating process of Li foil and Li-CNT electrodes. **(B)** Electrochemical impedance spectroscopy of Li||Li (left) and Li-CNT||Li (right) cells at different cycles (below: the equivalent circuit used for curve fitting). **(C)** Voltage profiles for stripping/plating cycles of Li-CNT||Li (red line) and Li||Li (black line) cells at different current densities (capacity is 20 mA h cm^−2^). **(D)** SEM images of the Li foil electrodes and the Li-CNT composite electrodes before and after short-circuit. Reprinted with permission from Wang Y. et al. (2017). Copyright (2017) Royal Society of Chemistry.

Combining Li metal with CNTs to form a Li-CNT electrode can properly guide the Li^+^ plating, alleviating the volume expansion and inhibiting dendrite growth (Wang Y. et al., 2017). According to the latter report, not only can the Li-CNT composite electrode accommodate the small-sized Li metal, but it can also maintain the shape of the carbon nanotube frame. Compared with pure lithium foil, a Li-CNT electrode is beneficial for reducing the probability of lithium dendrite formation and has a superior coulombic efficiency (CE). Many articles have reported that the CE directly obtained by the electrode during the plating/stripping process is not well used for pre-storing lithium (Ding et al., 2013; Lin et al., 2016; Liu et al., 2016). However, it is undeniable that Li-CNT electrodes can effectively inhibit dendrite growth (Figure 5A). At current densities of 0.1 and 0.5 mA cm^−2^, the overpotential of the pure lithium foil electrode shows a trend from large to small and finally stabilizes, while the overpotential of the Li-CNT composite electrode is very stable (Figure 5C). The initial dendrite growth causes the surface of the lithium foil to become uneven, increasing the specific surface area of the lithium metal and resulting in a decrease in interface resistance, which corresponds to the results of electrochemical impedance spectroscopy (Figure 5B). From the SEM images, one can observe that the lithium foil is almost entirely destroyed and the Li-CNT composite electrode still preserves its original morphology after cycling (Figure 5D). Therefore, CNTs play a significant role in lithium metal protection.

#### Graphene

**Figure 6:**
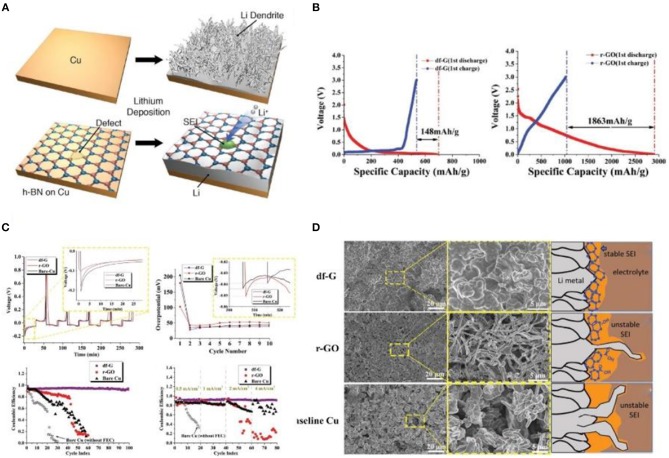
**(A)** Schematic diagrams of lithium deposition. Reproduced from Yan et al. (2014). Copyright (2014) American Chemical Society **(B)** Cycle 1 charge/discharge behavior of df-G and r-GO, respectively. Electrodes were tested in the high voltage range 3~0.01 V vs. Li/Li^+^, at 100 mA g^−1^. The lower voltage cutoff is significantly above the equilibrium Li plating potential of 0 V. **(C)** A comparison of the voltage profiles for df-G, r-GO, and bare Cu for the first several plating/stripping cycles at 0.5 mA cm^−2^, with the inset showing the cycle 1 (upper left corner) and cycle 10 (upper right corner) Li metal nucleation potential. Plating/stripping CE at 2 mA cm^−2^ and at various current densities, respectively. **(D)** Top-down SEM micrographs of the post-100-cycle electrode surfaces in the fully plated condition. Reprinted with permission from Liu et al. (2019). Copyright (2019) WILEY-VCH.

Graphene is a two-dimensional carbon nanomaterial composed of carbon atoms in an sp^2^ hybrid orbital hexagonal honeycomb crystal lattice. The chemical properties of graphene are similar to those of graphite, and it can adsorb and desorb various atoms and molecules. When these atoms or molecules act as donors or acceptors, the concentration of graphene carriers can be changed while the graphene itself maintains good conductivity. When other substances are adsorbed, such as H^+^ and OH^−^, some derivatives are produced that decrease the conductivity of graphene. Because graphene has good electrical conductivity, chemical stability in contact with electrolytes, and mechanical elasticity, it is widely used as a lithium storage medium by researchers (Xin-Bing et al., 2015; Kang et al., 2016; Lin et al., 2016; Zhang R. et al., 2016). Interestingly, Yan et al. (2014) grew two-dimensional (2D) atomic crystal layers, including hexagonal boron nitride (h-BN) and graphene, directly on Cu metal current collectors as an artificial SEI layer (Figure 6A). Moreover, using 3D monolithic corrugated graphene on nickel foam (CGNF) for a Li metal electrode suppresses dendrite growth in carbonate-based electrolytes (Kang et al., 2019). Recently, Chen and his team have found that defective graphene contributes to the growth of unstable SEI, resulting in an extremely unstable plating/stripping process (Liu et al., 2019). They adopted a new flow field-assisted ultrasonic stripping method to strip graphite into defect-free graphene (df-G) and obtained excellent electrochemical performance. They also adopted a test method for the negative electrode of LIBs that could effectively impede the undesirable interaction between the lithium metal and the electrolyte and restrict the initial formation of SEI in r-GO and df-G. The irreversible capacity loss in the first cycle for r-GO was 1,863 mA g^−1^, while that of df-G was only 148 mA h g^−1^ (Figure 6B). The SEI formation on the carbon surfaces with this initial capacity loss. In Figure 6C, the overpotential of the first lap of r-GO is lower than that of df-G, while as the cycling continues this trend is inverted. Therefore, df-G can suppress the formation of dendrites and maintain a high CE, low overpotential, and low nucleation barrier of lithium dendrites after plating/stripping 100 times at a current density of 2 mA cm^−2^. Furthermore, according to Figure 6D, df-G can restrain the Li dendrite growth and form a stable SEI on the surface of Li metal. It is well-known that nitrogen, oxygen, and silicon are pro-lithic, and many studies have looked into the relationship between the “lithiophilicity” of the template and the chemical nature of the carbon supports (Mukherjee et al., 2014; Zhang et al., 2017). However, some studies have found that pro-lithic groups are not necessarily beneficial. It has been reported that it is easier to grow a thicker SEI around nitrogen-doped carbon (Xu et al., 2017). In a nutshell, compared with carbon nanotubes, graphene is relatively difficult to migrate due to its lamellar structure. Therefore, researchers have improved the mobility of lithium ions in graphene by different methods to constrain the growth of dendrites.

#### Graphite

**Figure 7:**
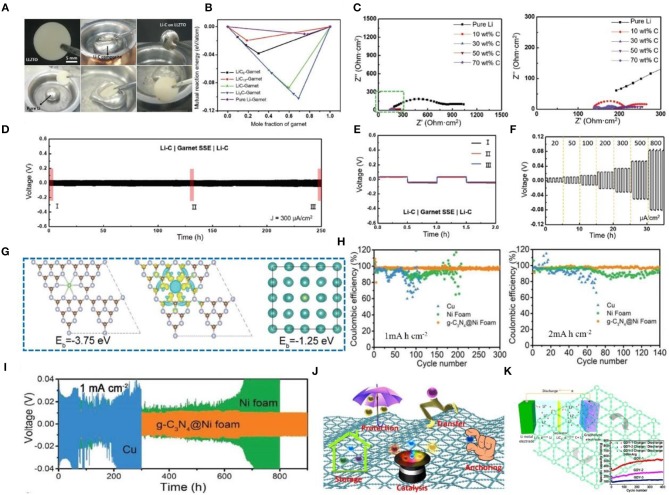
**(A)** Comparison between pure Li and Li-C lithophilic behaviors with a garnet-type LLZTO pellet. **(B)** Calculated mutual reaction energy of the Li-C composite/garnet interface and pure Li/garnet interface. **(C)** Nyquist plots of solid-state symmetric cells with pure Li electrodes and Li-C composite electrodes, where cells with Li-composite electrodes (carbon content from 10 to 70 wt%) show much smaller interfacial resistance. Reprinted with permission from Duan et al. (2019). Copyright (2019) WILEY-VCH. **(D)** Reversible plating/striping curves of symmetric-cell Li-C composite electrodes at 300 μA cm^−2^ and room temperature. **(E)** Three typical charge/discharge curves over long-term cycling as marked in **(D)**. **(F)** Rate performance of Li-C composite symmetric cells with step-ascending current density from 20 to 800 μA cm^−2^ at room temperature. Reprinted with permission from Duan et al. (2019). Copyright (2019) WILEY- VCH. **(G)** Crystal models for calculating the binding energy of Li^+^ adsorbed on g-C_3_N_4_; the corresponding charge density difference (brown, purple, and green balls represent carbon atoms, nitrogen atoms, and Li atoms, respectively; yellow and light blue areas represent positive and negative charge differences, respectively); crystal models for calculating the binding energy of Li^+^ adsorbed on Ni (blue and green balls represent nickel atoms and Li atoms, respectively). **(H)** Comparison of the CE of Li plating on/stripping from g-C_3_N_4_@Ni foam, Ni foam, and Cu electrodes with an areal capacity of 1.0 and 2.0 mA h cm^−2^ at the same current density of 2.0 mA cm^−2^. **(I)** Voltage-time profiles of Li plating/stripping with a cycling capacity of 1.0 mA h cm^−2^ at 1.0 mA cm^−2^ in a symmetrical cell. Reprinted with permission from Lu et al. (2019). Copyright (2019) WILEY-VCH. **(J)** Functions of GDY for electrochemical applications. Reprinted with permission from He et al. (2017). Copyright (2017) Springer Nature Limited. **(K)** Schematic illustration of a lithium-ion battery with GDY as the electrode and its cycling stability performance. Reprinted with permission from Huang et al. (2015). Copyright (2015) Elsevier Science BV.

Graphite belongs to the hexagonal system and possesses complete layered cleavage. The cleavage plane is mainly intermolecular. Graphite can be principally classified into several types as follows: (1) natural graphite, (2) artificial graphite (i.e., carbon fiber, pyrolytic carbon, foamed graphite, etc.), (3) bulk graphite, (4) flake graphite, and (5) cryptocrystalline graphite. Due to the good electrical conductivity, plasticity, and chemical stability of graphite, it is widely used in the field of Li metal anode protection. In a recent study, a lithium-graphite (Li-C) composite anode was fabricated that reduces the fluidity and increases the viscosity of Li metal and greatly improves the wettability of garnet solid-state electrolytes (Figure 7A) (Duan et al., 2019). According to first-principles calculation, Li-C composites can improve the interface affinity between Li and solid electrolytes (Figure 7B), and when the graphite content increases, the charge transfer resistance gradually decreases, delivering good cycle stability and rate performance (Figure 7C). We can see that the voltage hysteresis increases rapidly and the battery fails in a few hours. In comparison, a symmetric cell with a Li-C composite electrode shows long-term stability with a small voltage hysteresis over hundreds of hours (Figure 7D). Zoomed-in curves in Figure 7E provide further evidence of overlapped ultraflat plating/striping profiles during long-term cycling, indicating a stable interface between Li-C and garnet SSE. Apart from its stability, the Li-C/garnet SSE/Li-C cell also exhibits great rate capability. As shown in Figure 7F, the plating/striping curves are stable as the current density increases from 0.02 to 0.8 mA cm^−2^, indicating good cycle stability and rate performance. Lu et al. (2019) reported that graphitic carbon nitride (g-C_3_N_4_) coated onto foam nickel can form a three-dimensional current collector that can suppress the growth of Li dendrites and protect the Li metal anode. It is created by employing super nanosilver (Ag) particles as seeds in the deposition of Li in a three-dimensional host material, which results in uniform growth of Li (Kai Y. et al., 2016; Pei et al., 2017; Liu et al., 2018). Many studies have shown that local electric field changes can make Li deposition more uniform (Yang et al., 2015; Zou et al., 2018). The 3D current collector designed with a micro-electric field (MEF) by the author can make an even local electric field, leading to even Li^+^ deposition. DFT calculation can reveal the interaction and nucleation behavior of Li^+^ and g-C_3_N_4_. In this regard, g-C_3_N_4_ has the highest binding energy (−3.75 eV) with Li^+^, while Ni has a much smaller binding energy (−1.25 eV) (Figure 7G). The g-C_3_N_4_@Ni foam shows excellent CE (Figure 7H). Moreover, as shown in Figure 7I, the g-C_3_N_4_@Ni foam anode shows a smaller overpotential at a high amount of Li deposition (i.e., 9.0 mA h cm^−2^). In conclusion, the broad application of graphite is due to its good electrochemical performance, but its several shortcomings also limit its further application. Hence, many researchers are attempting to improve its performance through different methods, which is profoundly explored in later discussions.

#### Graphdiyne

The three hybrid states of carbon (i.e., sp, sp^2^, and sp^3^) are combined in various ways to produce different natural allotropes, such as graphite (sp^2^) and diamond (sp^3^) as well as many synthetic novel carbon allotropes such as fullerene (sp^2^), carbon nanotube (sp^2^), and graphene (sp^2^) (Kroto et al., 1985; Lijima, 1991; Novoselov et al., 2004). In recent years, scientists have been working to explore new forms of carbon allotropes. Accordingly, GDY was first synthesized by Li's group in 2010 (Guoxing et al., 2010), making it a new member of the graphite family. GDY has two hybrid states (sp and sp^2^) of carbon. Among the known carbon allotropes, GDY possesses the best mechanical strength and stiffness. Indeed, high conductivity and ion mobility, high chemical activity, and physical stability make GDY an ideal material for solving electrochemical interface problems. Meanwhile, GDY also shows a good affinity with metals (Figure 7J), making it possible to prepare flexible solid electrolytes (Zuo and Li, 2019). Its unique structure is more conducive to diffusion and transmission of Li^+^ in and out of the plane, ensuring a good rate performance. In terms of energy storage, GDY is extremely easy to grow on Si and metal oxide anodes (He et al., 2017), forming an all-carbon mechanically conductive interface, which is advantageous for high-speed transmission of electrons and ions (Shang et al., 2018).

More significantly, the high pore ratio and special structure of GDY provide large diffusion channels and storage sites for metal atoms, such as Li and Na (Huang et al., 2018). The range of lithiation potential of GDY is 2.7~2.1 V, and the intercalation density of lithium is LiC_6_ in graphite, and LiC_3_ in Zhang et al. (2011, 2013). In recent researches, Huang et al. (2015) and Zhang et al. (2015) demonstrated that employing GDY in LIBs (Figure 7K) yields superior performance compared to graphite. What is more, the defects on GDY and the introduction of N (Wang N. et al., 2017), and Cl (Zhang S. et al., 2016) atoms can increase energy storage sites, which is beneficial for reducing interfacial reactions and stabilizing the interface. Note that modification of GDY by doping is also beneficial to electrochemical performance improvement, which is a common modification method for graphene. In a word, GDY shows great prospects in Li metal protection owing to its superior performance (Zuo and Li, 2019). As one of the most commercially valuable materials in the future, GDY will play a huge role in many fields besides the field of electrochemistry.

### Doped Carbon Materials

Although carbon materials have achieved remarkable results in Li metal protection, there is still a lot of room for development. Currently, there are a variety of methods to modify carbon materials. For instance, lithophilic groups (N, Si, B, F, etc) and materials with solubility (Ag, Zn, Mg, Al, Pt, etc) can be introduced by doping, which can change the initial formation behavior of Li deposition and reduce the nucleation overpotential, thereby restraining the growth of dendrites from the beginning. Moreover, lithophilic groups can increase the specific surface area of the carbon material, decrease local current density, and reduce the nucleation size of Li.

**Figure 8:**
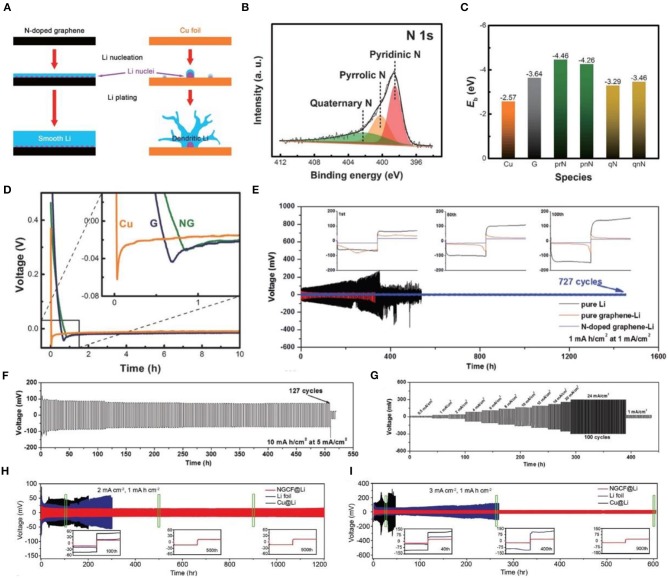
**(A)** Schematic representation of the Li nucleation and plating process on an N-doped graphene electrode and a Cu foil electrode. **(B)** N 1s XPS spectra of N-doped graphene. **(C)** Binding energy of a Li atom with Cu, graphene **(G)**, and different functional groups of N-doped graphene. **(D)** Nucleation overpotential and the voltage-time curves during Li nucleation at 0.05 mA cm^−2^ on Cu foil, G, and NG electrodes. Reprinted with permission from Zhang et al. (2017). Copyright (2017) WILEY-VCH. Galvanostatic cycling of pure Li foil, pure graphene-Li, and N-doped graphene-Li symmetric cells with a stripping/plating capacity of 1 mA h cm^−2^ at a current density of 1 mA cm^−2^. Insets in **(E)** are the representative voltage profiles. **(F)** Galvanostatic cycling of symmetric N-doped graphene-Li cells with a stripping/plating capacity of 10 mA h cm^−2^ at a current density of 5 mA cm^−2^. **(G)** Rate performance of N-doped graphene-Li electrodes measured at current densities ranging from 0.5 to 12 mA cm^−2^ for 1 h and from 16 to 24 mA cm^−2^ at an area capacity of 12 mA h cm^−2^ in both the stripping/plating processes of each cycle. Reprinted with permission from Huang et al. (2019). Copyright (2019) WILEY-VCH. **(H)** Voltage profiles of metallic Li plating/stripping in three styles of symmetric cells (NGCF@Li, Li foil, and Cu@Li cells) at 2 mA cm^−2^ for 1 mA h cm^−2^. Inset: magnified Li plating/stripping profiles in the 100th, 500th, and 900th cycles, respectively. **(I)** Voltage profiles of metallic Li plating/stripping in three styles of symmetric cells at 3 mA cm^−2^ for 1 mA h cm^−2^. Inset: Magnified Li plating/stripping profiles in the 40th, 400th, and 900th cycles, respectively. Reprinted with permission from Liu et al. (2018). Copyright (2018) WILEY-VCH.

Zhang et al. (2017) used N-doped graphene (NG) to achieve uniform nucleation of Li. Figure 8A presents the nucleation and electroplating process of metallic lithium on N-doped graphene-based bodies and copper current collectors. The N-containing functional groups in NG, including pyridinic N, pyrrolic N, and quaternary N (Figure 8B), are beneficial to metallic Li nucleation, leading to an even distribution of metal on the surface of the anode (Zhang et al., 2017). According to DFT calculations (Figure 8C), there is a weak interaction between lithium and a copper current collector, while the interaction between pyridine and pyrrole N is very strong, which is consistent with previous assumptions. In addition, electron donor-rich NG possesses an additional electron pair. When the P orbital is filled, it can be considered as a Lewis base, which interacts with the Lewis acid Li^+^ in the electrolyte to cause uniform deposition on the lithium surface (Hou et al., 2016). Additionally, it can be seen from the voltage-time curve (Figure 8D) that the nucleation overpotential of NG is the lowest (0.022 V), while that of Cu is the highest (0.053 V). Thus, NG can guarantee a dendrite-free lithium metal anode. However, traditional porous carbon hosts, such as porous carbon nanotubes, and porous graphene provide greater advantages than do porous metals (Zheng et al., 2014; Lin et al., 2016; Zhang R. et al., 2016; Jin et al., 2017; Wang T. et al., 2017; Ye et al., 2017), and the limited surface area cannot effectively attenuate the rate performance of the Li metal anode under high current densities (Zhang R. et al., 2016; Jin et al., 2017; Raji et al., 2017). In recent reports, a nanoporous N-doped graphene-reinforced Li anode (Huang et al., 2019) was applied for Li metal protection. From charging and discharging tests on symmetrical batteries assembled of pure Li, pure graphene-Li, and N-doped graphene (Figure 8E), one can observe that the voltage hysteresis of the N-doped graphene electrode is much smaller than those of the other two electrodes and remains stable after 727 cycles without any obvious voltage fluctuations. It is also evident from the enlarged time-voltage relationship diagram that there are significant differences between the three electrodes. N-doped graphene is significantly better than the other two electrodes. Additionally, it is found that when the deposition capacity and current density are increased, the voltage hysteresis of the N-doped graphene electrode is not meaningful, but the dendrite growth is severe after 127 cycles, piercing the separator, leading to battery short-circuit (Figure 8F). The rate performance test demonstrates that when the current density range is wide, the polarization of the electrode is weakened (Figure 8G). Suffice it to say, owing to the large specific surface area and high conductivity of N-doped graphene, it shows great prospects in LMBs.

Furthermore, a self-supporting carbon film (Chen et al., 2019) consisting of a nitrogen (N)-doped carbon rod array was developed for lithium metal protection and achieved remarkable results. N-doped graphitic carbon foams (NGCFs) were also synthesized to suppress dendritic Li growth at the nucleating stage (Liu et al., 2018). According to the time-voltage profiles in Figure 8H, the NGCF@Li symmetrical cell at 2 mA cm^−2^ with 1 mA h cm^−2^ exhibits an extremely stable voltage and a long cycle life (1,200 h), which is superior to the Li foil and Cu@Li symmetrical cell. When the current density is increased to 3 mA cm^−2^ (Figure 8I), the same result is obtained (the NGCF@Li symmetrical cell exhibits stable cycling for 600 h). At present, N-doped modified carbon materials are a research hotspot in the field of electrochemistry.

**Figure 9:**
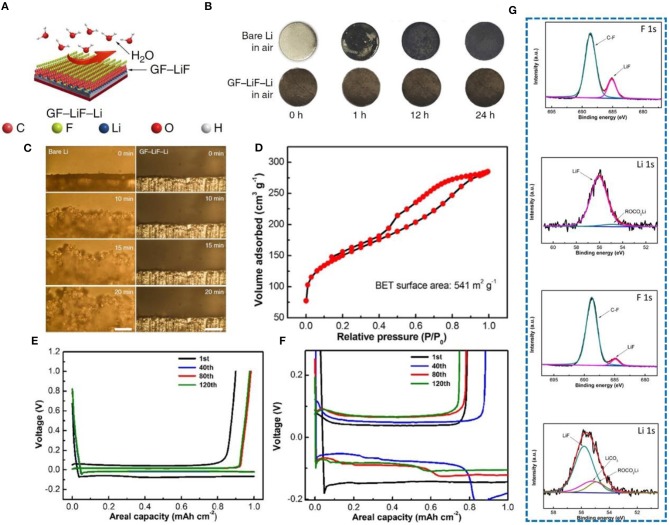
**(A)** Schematic illustration of the GF-LiF-Li protective effect for Li-metal anodes. In the models, the carbon (C), fluorine (F), lithium (Li), oxygen (O), and hydrogen (H) atoms are displayed as orange, cyan, blue, red, and white spheres, respectively. **(B)** Characterization of GF-LiF-Li air stability. **(C)** *In-situ* optical microscopy visualization of Li electrodeposition and long-term cycling on symmetric cells. Images from microscopy of the bare Li (left column) and GF-LiF-Li (right column) electrolyte interface at 0, 10, 15, and 20 min at a current rate of 3 mA cm^−2^. The scale bars are 200 μm. Reprinted with permission from Shen et al. (2019). Copyright (2019) Springer Nature Limited. **(D)** Nitrogen adsorption-desorption isotherms. **(E)** Voltage profiles of MGCN for the annotated cycles. **(F)** Voltage profiles of Cu foam for the annotated cycles. **(G)** XPS graph of LiF-rich SEI, F 1s, and Li 1s XPS spectra of the SEI layer from MGCN and Cu foam electrodes after Li stripping. The results reveal the formation of a LiF-enriched SEI layer on the MGCN electrode upon initial electrochemical lithiation. Reprinted with permission from Li et al. (2018). Copyright (2018) Elsevier Science BV.

On the basis of N doping, the performance of F-doped carbon materials has also been investigated. Lately, employing graphite fluoride (GF) (Lazar et al., 2015; Wang et al., 2015) to form a hydrophobic solid-electrolyte interphase on a metallic-lithium anode has been explored (Shen et al., 2019). This special anode was prepared by adding graphite fluoride into molten Li, forming a sandwich structure (Figure 9A). In order to prove its hydrophobicity, the Li sheet and GF-LiF-Li composite anode were exposed to the air, and surface changes were carefully observed. After 24 h, the surface of the Li sheet changed significantly, but the composite electrode did not change significantly (Figure 9B). Therefore, it was proved that a GF-LiF-Li composite anode has good stability in the atmosphere. The deposition process of Li at different times between a pure Li electrode and a GF-LiF-Li electrode was observed by *in-situ* optical microscopy (Figure 9C), and severe growth of dendrites on the pure Li electrode is clearly noticeable, while no dendrites can be observed on the GF-LiF-Li electrode surface. Thus, the GF-LiF-Li composite anode can suppress dendrite growth to a large extent, providing an opportunity for assembling LMBs in the atmosphere, which substantially reduces production cost. Li et al. (2018) constructed a self-supporting three-dimensional fluorine-doped graphene shuttle-implanted porous carbon network (MGCN) as a multifunctional host matrix for lithium to inhibit dendrite growth. The addition of graphene can increase the electronic conductivity of MGCN. Its three-dimensional porous structure can improve the mobility of lithium ions. Figure 9D shows that MGCN has a high specific surface area. The voltage distribution profiles of MGCN and of a Cu foam symmetrical cell were used to verify the structural advantages of MGCN. The electroplating nucleation potential of Li on MGCN is <5 mV (Figure 9E), while that on Cu foam is more than 40 mv (Figure 9F). Fluorine doping leads to a uniform distribution of lithium ions and the formation of a LiF-rich SEI (proved by XPS test shown in Figure 9G), which is one of the most effective methods for inhibiting dendrite growth.

**Figure 10:**
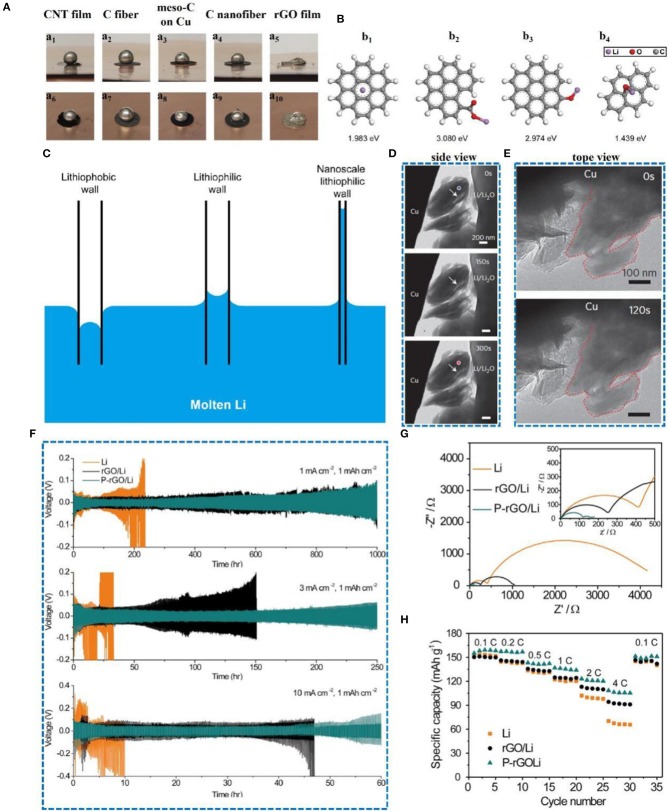
**(A)** Lithiophilicity of various carbon materials. Surface wetting of molten Li on different carbon materials, including CNT film (a_1_, a_6_), carbon fiber paper (a_2_, a_7_), mesoporous carbon coated on Cu foil (a_3_, a_8_), carbon nanofiber (a_4_, a_9_), and sparked-rGO film (a_5_, a_10_). For sparked-rGO film, the molten Li was rapidly infused into the matrix, with good wettability. In contrast, the other carbon materials showed relatively large contact angles, indicating relatively poor Li surface wettability. **(B)** First-principles calculations showing the binding energy between Li and a bare graphene surface (b_1_), carbonyl (C = O) groups (b_2_), alkoxy groups (C-O) (b_3_), and epoxyl (C-O-C) groups (b_4_). The carbonyl and alkoxy groups show much stronger interaction with Li compared to a bare graphene surface. **(C)** Capillary force at different scales and lithiophilicities. **(D)** Time-lapse images showing a side view of an rGO film during the Li deposition process. **(E)** Top view of an rGO film at the initial (top) and final (bottom) stages of Li deposition. Reprinted with permission from Lin et al. (2016). Copyright (2016) Springer Nature Limited. **(F)** Symmetric cell-cycling performance of Li foil, rGO/Li, and P-rGO/Li under 1, 3, and 10 mA cm^−2^. The areal capacity was fixed at 1 mA h cm^−2^. **(G)** Electrochemical impedance spectroscopy of symmetric cells of Li foil, rGO/Li, and P-rGO/Li before electrochemical testing. **(H)** Rate capability of LFP|Li, LFP|rGO/Li, and LFP|P-rGO/Li cells from 0.2 to 3 C. The areal capacities of LFP cathodes and anodes used here were ~2 and 10 mA h cm^−2^, respectively. Reprinted with permission from Wang et al. (2018). Copyright (2018) Elsevier Science BV.

Oxygen-doped carbon materials have also been widely employed. A Li-rGO electrode was formed by injecting molten Li into a layer of rGO film (Lin et al., 2016), which significantly inhibited the growth of dendrites. It was also found that the molten Li was in contact with the rGO film and injected into the rGO at a very fast rate, which was unprecedented among all of the current carbon materials (Figure 10A). The surface of rGO is rich in carbonyl and alkoxy groups (carbonyl is 3.080 eV, alkoxy is 2.974 eV), so the binding energy between Li and rGO is much larger (Figure 10B) than that of graphene, which why rGO can absorb the molten Li so quickly. Therefore, rGO has an excellent lithiophilicity, which is related to its scale; the smaller the size, the larger the capillary force (Figure 10C). Figures 10D,E show that during the deposition of lithium, the thickness and volume barely change and no dendrites are visible on the surface. Thus, the rGO film can adjust the volume expansion of Li metal and guide the plating of lithium. Recently, a patterned reduced graphene oxide (P-rGO) /Li anode was also developed (Zou et al., 2018). The electric field around the edge of the patterned electrode can direct lithium ions according to the electric field direction. The voltage profiles of the symmetrical cells were tested at 1, 3, and 10 mA cm^−2^ with 1 mA h cm^−2^. The P-rGO/Li anode shows a stable cycle life and relatively low voltage hysteresis compared to metallic lithium and reduced graphene oxide (Figure 10F). The corresponding impedance and rate performance test results are also consistent with the expected results (Figures 10G,H). Additionally, based on the excellent lithiophilic performance and mechanical strength of reduced graphene oxide, Yu's team developed (Yu et al., 2019) a reduced graphene oxide paper to adjust the electroplating process of lithium, and the rapid diffusion of lithium ions was successfully achieved.

### Composites With Carbon Materials

**Figure 11:**
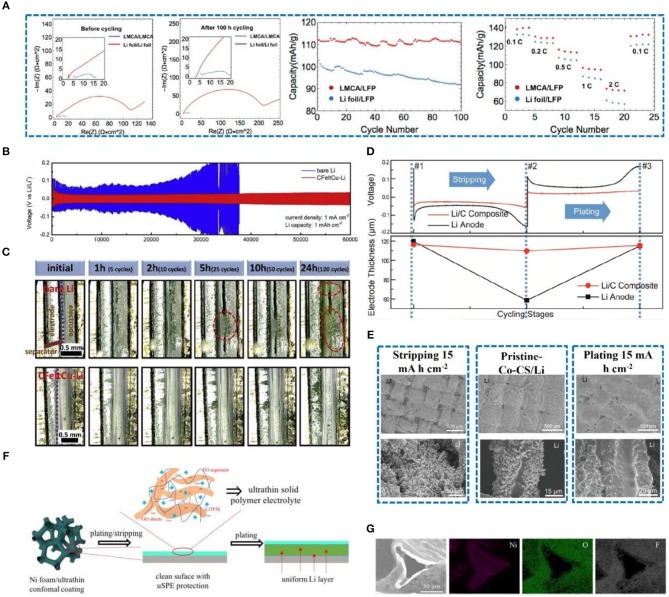
**(A)** EIS results for LMCA/LMCA and Li foil/Li foil cells at 0.5 mA cm^−2^ before cycling, EIS results for LMCA/LMCA and Li foil/Li foil cells after 100 h of cycling at 0.5 mA cm^−2^, long-term cycling of LMCA/LFP and Li foil/LFP full cells at 0.5 mA cm^−2^ current density with 2.6 and 3.8 V voltage cutoffs, and rate capability test of LMCA/LFP and Li foil/LFP full cells at different current densities with 2.6 and 3.8 V voltage cutoffs. Reprinted with permission from Li et al. (2019a). Copyright (2019a) American Chemical Society. **(B)** Voltage profile of bare Li and CFeltCu-Li electrodes cycled at 1 mA cm^−2^ with a capacity of 1 mA h cm^−2^. **(C)** Cross-sectional *in-situ* electron optical microscopy images of the bare Li and CFeltCu-Li electrodes during the cycles at a current density of 10 mA cm^−2^ (capacity is 1 mA h cm^−2^). Reprinted with permission from Yue et al. (2019). Copyright (2019) Elsevier Science BV. **(D)** Voltage profile of a typical Li-stripping-plating process for the anode and its corresponding thickness at various stages of cycling. #1 corresponds to the stage before cycling; #2 corresponds to the stage after ~50% Li extraction (10 mA h cm^−2^); #3 corresponds to the stage after Li plating back. Reprinted with permission from Liang et al. (2016). Copyright (2016) PANS. **(E)** Low-magnitude SEM images of a Li/Co-CS anode after stripping and plating at 15 mA h cm^−2^ Li, and high magnitude SEM images of a single carbon fiber at the different Li deposition periods. Reprinted with permission from Li et al. (2019b). Copyright (2019b) WILEY-VCH. **(F)** Schematic of the stripping/plating behavior of Li Ni foam with an ultrathin conformal coating. **(G)** SEM image of Ni foam/uSPE and corresponding elemental mapping of Ni, O, and F. Reprinted with permission from Deng W. et al. (2019). Copyright (2019) Royal Society of Chemistry.

As previously mentioned, carbon-based materials have been widely used in lithium metal protection due to their good electrical conductivity, thermal conductivity, and chemical stability. At present, combining carbon-based materials with other materials to form composites is an effective strategy for lithium metal protection. In this regard, a freestanding porous lithium metal microparticle (Li MP)/carbon nanotube composite anode (LMCA) was prepared (Li et al., 2019a). The electrochemical performance of the half-cell and full-cell assembled with LMCA is significantly better than that with Li foil (Figure 11A). The three-dimensional porous structure of LMCA increases the specific surface area, reduces current density, and alleviates volume expansion. Moreover, a 3D Cu-CuO-Ni conductor (Wu et al., 2018), 3D nitrogen-doped graphene foams (Liu et al., 2018), and a 3D porous Cu collector (Li et al., 2017) have also been employed to protect the Li metal anode. However, the framework has not fully met the requirements, i.e., it is either too heavy (Chi et al., 2017) or has unsatisfactory lithiophilicity. Recently, a three-dimensional carbon felt (CFelt) coated with a copper oxide layer was synthesized (Yue et al., 2019) that can effectively inhibit dendrite growth and improve the electrochemical performance of the batteries. The 3D CFelt conductive skeleton has good flexibility and excellent electrical conductivity, allowing it to perfectly alleviate volume expansion, reduce local current density, and improve Li stripping/plating ability (Chen et al., 2017). Cu nanoparticles can further reduce current density and synergize with conductive carbon felt. Therefore, as shown in Figure 11B, a CFeltCu-Li anode has a low voltage hysteresis and long cycle life compared with a pure Li anode, which attributes to the CFeltCu with preferable lithiophilicity that can reduce the nucleation overpotential of Li and impede the formation of dendrites from the beginning. Besides, as depicted in Figure 11C, as time passes, the accumulation of “dead Li” significantly increases the thickness of the bare lithium. On the contrary, there is no “dead Li” accumulation and consequently no change of thickness on the CFeltCu-Li electrode.

Liang et al. (2016) manufactured a stable composite electrode that was prepared by pouring molten lithium into a 3D porous carbon matrix. The Si-coated porous object can stabilize the electrode/electrolyte interface, reduce the overpotential of Li nucleation, increase the surface area, decrease the local current density, and improve the electrochemical performance. Moreover, a Li/C anode has excellent interface stability during cycling (Duan et al., 2013; Kang et al., 2015), and the overpotential is relatively small compared with a pure Li anode (Figure 11D). The Li/C anode can greatly alleviate the volume expansion of Li, which plays a positive role in suppressing dendrite growth. Moreover, a Co_3_O_4_ nanofiber carbon sheet (Co_3_O_4_-CS) skeleton with excellent pro-sodium/pro-lithium performance was reported by Li et al. (2019b) that can act as a stable matrix for an alkali metal electrode. They revealed that the pro-lithium performance of metal oxides has a relationship with the thermodynamics of chemical reactions and thus that Co_3_O_4_-CS induces good lithiophilicity and uniform nucleation. After stripping and plating, the carbon fiber structure can be clearly observed in the SEM images. The surface of the carbon fiber is completely adhered to the Li (Figure 11E), thereby confirming that Li^+^ is uniformly deposited on the surface of the Co_3_O_4_-CS/Li electrode. This innovative method can effectively improve the cycle stability of a Li metal anode and inhibit the appearance of dendrites.

Although the 3D porous structure has a large specific surface area and effectively stores Li to alleviate the volume expansion of Li metal, the large specific surface area means that the contact area with the electrolyte is also increased (Liang et al., 2017), and the irreversible reaction on the electrode is amplified. This not only causes a degradation in electrolyte performance but also produces products such as Li_2_CO_3_, lithium alkyl carbonate, and alkyl lithium decomposition, covering the electrode surface and hindering further electron/mass transfer (Lee et al., 2013; Deng et al., 2018a; Jiao et al., 2018). The above-mentioned phenomenon has been reported in many studies (Deng et al., 2018b; Wu et al., 2018; Zhang R. et al., 2018). Recently, a surface-engineered coating was developed (Deng W. et al., 2019) with solid polymer electrolyte (SPE) containing polyethylene oxide (PEO) as the polymer matrix, lithium bis (trifluoromethanesulfonyl) imide (LiTFSI) as the Li salt, and graphene oxide (GO) sheets as an enhancing filler (Figure 11F). The ultrathin and conformal SPE coating layer (uSPE) on Ni foam presents a large charge transfer resistance (0.5 Ω) compared to pure Ni foam (0.375 Ω), which can affect the migration of lithium ions. The uniform distribution of O and F on the surface of the Ni foam proves the smoothness of the uSPE coating (Figure 11G). This is consistent with the original conjecture that evenly distributed the uSPE coating allows Li^+^ to conduct quickly while avoiding electrolyte contact with metallic lithium, reducing the probability of electrolyte decomposition and side reactions.

Notably, researchers have only focused on the large specific surface area of the 3D current collector, which greatly alleviates the volume expansion of metallic lithium, and have ignored its drawbacks. It is worth mentioning that the large specific surface area increases the probability of contact between metallic lithium and the electrolyte and subsequently increases irreversible interfacial reaction of the electrode/electrolyte and has a negative effect on preventing dendrite growth. Accordingly, it is necessary to modify the surface of the 3D current collectors via other approaches.

## The Application of Lithium Metal Anodes

**Figure 12:**
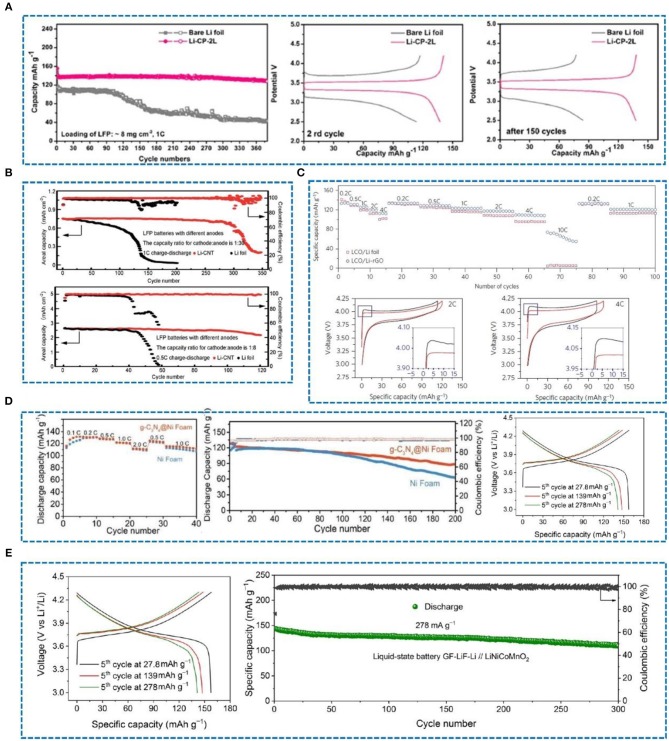
**(A)** Cycling performance of full cells (C/LiFePO_4_ as the cathode) using bare Li foil and Li-CP-2L at 1 C, and voltage hysteresis profiles of charge/discharge of bare Li foil and Li-CP-2L in the 2nd cycle and after the 150th cycle. Reprinted with permission from Zhao et al. (2018). Copyright (2018) Elsevier Science BV. **(B)** The capacity ratio of cathode to anode is 1:30, and the capacity ratio of cathode to anode is 1:8. Reprinted with permission from Wang Y. et al. (2017). Copyright (2017) Royal Society of Chemistry. **(C)** Rate capability of LCO/Li-rGO and LCO/Li foil cells at various rates from 0.2 C to10 C, and voltage profile comparison of the LCO/Li-rGO cells at rates of 2 C and 4 C. Reprinted with permission from Lin et al. (2016). Copyright (2016) Springer Nature Limited. **(D)** Rate performance of Li@g-C_3_N_4_@Ni foam|LiCoO_2_ and Li@Ni foam|LiCoO_2_ cells, discharge capacity and CE of Li@g-C_3_N_4_@Ni foam|LiCoO_2_ and Li@Ni foam|LiCoO_2_ cells at 1.0 C, and charge-discharge profiles of full cells with LiCoO_2_ as the cathode and Li@g-C_3_N_4_@Ni foam (2 mA h cm^−2^ Li deposition) as the anode at 1.0 C. Reprinted with permission from Lu et al. (2019). Copyright (2019) WILEY-VCH. **(E)** The characteristic charge-discharge voltage profiles of liquid-state GF-LiF-Li//LiNiCoMnO_2_ cells at different current densities, and a long-term cycling test of GF-LiF-Li//LiNiCoMnO_2_ cells at a current density of 278 mA g^−1^. Reprinted with permission from Shen et al. (2019). Copyright (2019) Springer Nature Limited.

Carbon-based materials are widely used in lithium metal protection owing to their superior thermal and electrical conductivity, high temperature resistance, good mechanical strength, excellent chemical stability, and good lubrication performance. As a result, carbon-based materials can efficiently reinforce materials lithiophilicity, increase lithium ion mobility and ease the volume expansion of lithium metal. The purpose of studying the protection of lithium metal anodes is to pair them with a variety of cathodes and then assemble them into full batteries. Zhao et al. (2018) have investigated carbon paper (CP) as an interlayer to protect lithium metal anodes, which was paired with a LiFePO_4_(LFP) cathode to form a full battery. After 380 cycles, compared with the pure Li foil, the capacity can be stabilized at about 130 mA h g^−1^. Indeed, in the 2nd cycle and 150th cycle, it shows a small voltage hysteresis (Figure 12A). As shown in Figure 12B, when the Li foil and the Li-CNT electrode are paired with a commercial LFP cathode, the CE can be stabilized at 90.0% and 90.1% after 100 and 300 cycles at 0.5 C and 1 C, respectively (Wang Y. et al., 2017). Moreover, in a full cell with Li-reduced graphene oxide (rGO) as the anode and LiCoO_2_ as the cathode (Lin et al., 2016), the battery maintains a high capacity at high rates, especially at 10 C, and shows a small overpotential at different rates (Figure 12C) compared with pure Li foil. In another study, a Li@g-C_3_N_4_@Ni foam and Li@Ni foam composite anodes were paired with a LiCoO_2_ cathode. The Li@g-C_3_N_4_@Ni foam|LiCoO_2_ cell exhibited a stable capacity (Figure 12D) and delivered 72.9% capacity retention (Lu et al., 2019). In a recent article, a GF-LiF-Li anode with good stability in an atmospheric environment was paired with a LiNiCoMnO_2_ cathode (Shen et al., 2019) and showed a normal charge-discharge curve (Figure 12E). CE was close to 100%, and the capacity fading rate was very low, about 0.06% per cycle after 300 cycles.

**Figure 13:**
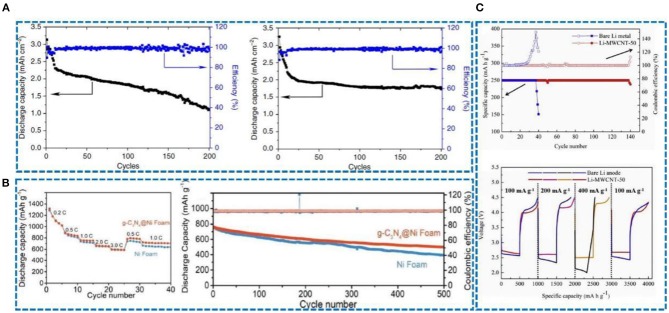
**(A)** Discharge capacity and coulombic efficiency of Li-S batteries with a pristine Li foil, and discharge capacity and coulombic efficiency of Li-S batteries with a GZCNT-coated Li foil anode. Reprinted with permission from Zhang H. et al. (2018). Copyright (2018) Springer Nature Limited. **(B)** Rate performance of Li@g-C3N4@Ni foam|S and Li@Ni foam|S cells (1.0 C = 1,675 mA g^−1^ for a sulfur cathode), and discharge capacity and CE of Li@g-C3N4@Ni foam|S and Li@Ni foam|S cells at 1.0 C. Reprinted with permission from Lu et al. (2019). Copyright (2019) WILEY-VCH. **(C)** Cycling stability of the Li-O_2_ batteries with and without an MWCNT interlayer at a fixed current density of 100 mA g^−1^ and capacity of 250 mA h g^−1^ from 2.0 to 4.5 V, and rate capability performances of the Li-O_2_ batteries with and without an MWCNT interlayer at increased current density. Reprinted with permission from Deng Y. et al. (2019). Copyright (2019) Elsevier Science BV.

In another study, the author developed a lithiophilic-lithiophobic gradient strategy by dripping carbon nanotubes (CNT), with various ZnO loadings, layer by layer onto Li foil (GZCNT) (Zhang H. et al., 2018). Li-S batteries were assembled to examine the performance of the GZCNT-coated Li anode. The cycle stability of GZCNT was significantly improved at 0.2 C. After 200 cycles, the remaining capacity of the GZCNT battery was 1.73 mA h cm^−2^ (Figure 13A), which is higher than that of the Li foil (1.12 mA h cm^−2^). Hence, a Li foil anode with GZCNT coating is extremely effective for improving the electrochemical performance of Li-S batteries. As shown in Figure 13B, Li@g-C_3_N_4_@Ni foam|S cells were analyzed to verify their potential use in full cells and showed good rate performance and capacity retention (Lu et al., 2019). In a recent study, multi-walled carbon nanotubes (MWCNT) were used as an interlayer in combination with lithium metal as a negative electrode (Deng Y. et al., 2019) and assembled into a Li-O_2_ battery to verify the possibility of practical application. Compared with a pure Li electrode, the Li-MWCNT electrode delivers a more stable CE after about 140 cycles (Figure 13C) and has an excellent rate performance. It exhibits a lower voltage hysteresis than bare Li at all current densities.

## Conclusion and Perspective

In recent years, low-capacity graphite anodes have no longer been able to meet the needs of human life, and it has become urgent to find appropriate high-capacity anode materials. Hence, a research boom has occurred on the topic of lithium metal anodes. The modification of metallic lithium with a carbon material, in particular, has achieved satisfactory results. Practically, many results have been obtained under laboratory conditions, which have huge prospects for Li-S and Li-O_2_ batteries in particular. Carbon-based materials possess high conductivity, good chemical stability, and low price and can be used as a good host matrix for accommodating lithium volume expansion and achieving a lighter anode. Although significant effects have been achieved, the inherent instability of metallic lithium in organic electrolytes still needs to be solved urgently. Carbon materials have their own limitations. For instance, their affinity with lithium is not as good as those of metal, metal oxide, and metal sulfide materials, which is a big challenge. In future work, it is inevitable that carbon materials will be utilized in lithium metal protection and that their lithiophilicity will be improved. Due to the complicated internal conditions of the batteries, the mechanism of action for carbon materials is still unclear. In addition, Li-S, Li-O_2_, and all-solid-state lithium batteries are considered to be the most promising next-generation energy storage devices. However, there are some issues that need to be resolved first: (1) the poor electrical conductivity of sulfur, and the lithium polysulfide shuttle effect in Li-S batteries, (2) the need for a catalyst for cathode reactions, electrolyte evaporation, electrolyte oxidation, the reactions of moisture and CO_2_ with lithium metal, and air electrode hole blockage in Li-O_2_ batteries, (3) the low ionic conductivity of solid electrolytes and poor contact with metallic lithium in all-solid-state lithium batteries. Therefore, multi-faceted research on carbon materials will be very helpful for improving LMB performance.

## Author Contributions

All authors wrote the manuscript. XL and SL supervised the manuscript.

### Conflict of Interest

The authors declare that the research was conducted in the absence of any commercial or financial relationships that could be construed as a potential conflict of interest.
